# Bioimpedanciometry in nutritional and hydration assessments in a single dialysis center

**DOI:** 10.1590/2175-8239-JBN-2022-0037en

**Published:** 2022-12-05

**Authors:** Claudia Zeni, Gisele Meinerz, Roger Kist, Catarina Bertaso Andreatta Gottschall, Brunno Brochado Jorge, João Carlos Goldani, Elizete Keitel

**Affiliations:** 1 Santa Casa de Misericórdia de Porto Alegre, Departamento de Nefrologia e Transplante de Rim e Pâncreas, Porto Alegre, RS, Brazil.; 2 Universidade Federal de Ciências da Saúde de Porto Alegre, Programa de Pós-Graduação em Patologia, Porto Alegre, RS, Brazil.; 3 Universidade Federal de Ciências da Saúde de Porto Alegre, Programa de Pós-Graduação em Ciências da Saúde, Porto Alegre, RS, Brazil.; 4 Universidade Federal de Ciências da Saúde de Porto Alegre, Programa de Graduação em Nutrição, Porto Alegre, RS, Brazil.; 5 Universidade Federal de Ciências da Saúde de Porto Alegre, Programa de Iniciação Científica, Porto Alegre, RS, Brazil.

**Keywords:** Electric Impedanc, Renal Dialysi, Body Compositio, Mortality, Impedância Elétrica, Diálise Renal, Composição Corporal, Mortalidade

## Abstract

**Background:**

Bioimpedance analysis (BIA) has been demonstrated to add accuracy to nutritional and volume status assessments in dialysis (HD) patients.

**Aim:**

to describe a sample of dialysis patients from a single center on their demographics and BIA of volume distribution and nutritional status, and mortality during 12-month follow-up.

**Methods:**

prospective observational cohort study to evaluate vintage HD patients with single-frequency BIA.

**Results:**

we evaluated 82 patients, 29% over 65 years old. Elderly patients had higher ECW/TBW (0.51 vs. 0.44, p < 0.0001), and narrower phase angle (PhA) (4.9 vs. 6.4º, p < 0.0001). Fifteen patients (18.2%) died during follow-up, eight (53%) were elderly. Death was associated with age (62.6 vs. 50.2 years, p = 0.012), post-HD PhA (4.8 vs. 6.2º, p = 0.0001), and post-HD ECW/TBW (0.50 vs. 0.45, p = 0.015). The ROC curve analysis to predict mortality found ECW/TBW ≥ 0.47 and PhA ≤ 5.5º to have the best sensitivity and specificity. One-year patient survival was lower with post-HD ECW/TBW ≥ 0.47 (69.5% vs. 90.6%, p = 0.019), age ≥ 65 years (64.2%, vs. 86.2%, p = 0.029), and PhA ≤ 5.5º (68.2 vs. 91.0%, p = 0.002). Cox regression analysis demonstrated that PhA [HR 5.04 (95%CI 1.60–15.86), p = 0.006] remained associated with death after adjusting for age and ECW/TBW.

**Conclusion:**

BIA is useful in assessing volume distribution and nutrition in HD patients, and combined with clinical judgement, may help determine dry weight, especially in elderly patients. Narrower PhA and higher ECW/TBW after HD were associated with poorer one-year survival.

## RESUMO

**Antecedentes:**

Análise de bioimpedância (BIA) demonstrou adicionar acurácia às avaliações de estado nutricional e de volume em pacientes em diálise (HD).

**Objetivo:**

descrever amostra de pacientes em diálise de um único centro quanto aos aspectos demográficos e BIA na distribuição de volume e no estado nutricional, e a mortalidade em 12 meses de acompanhamento.

**Métodos:**

estudo de coorte observacional prospectivo para avaliar pacientes prevalentes em HD com BIA de frequência única.

**Resultados:**

avaliamos 82 pacientes, 29% acima de 65 anos. Pacientes idosos apresentaram maior AEC/ACT (0,51 vs. 0,44; p < 0,0001), e ângulo de fase mais estreito (PhA) (4,9 vs. 6,4º; p < 0,0001). Quinze pacientes (18,2%) foram a óbito durante acompanhamento, oito (53%) eram idosos. Óbito foi associado à idade (62,6 vs. 50,2 anos, p = 0,012), PhA pós-HD (4,8 vs. 6,2º; p = 0,0001), e AEC/ACT pós-HD (0,50 vs. 0,45, p = 0,015). A análise da curva ROC para prever mortalidade constatou que AEC/ACT ≥ 0,47 e PhA ≤ 5,5º apresentam melhor sensibilidade e especificidade. Sobrevida do paciente em um ano foi menor com AEC/ACT pós-HD ≥ 0,47 (69,5% vs. 90,6%; p = 0,019), idade ≥ 65 anos (64,2% vs. 86,2%; p = 0,029), e PhA ≤ 5,5º (68,2 vs. 91,0%; p = 0,002). A análise de regressão de Cox demonstrou que PhA [HR 5,04 (IC 95% 1,60–15,86); p = 0,006] permaneceu associado ao óbito após ajuste para idade e AEC/ACT.

**Conclusão:**

BIA é útil ao avaliar distribuição de volume e nutrição em pacientes em HD, e juntamente com julgamento clínico, pode ajudar a determinar o peso seco, principalmente em pacientes idosos. PhA mais estreito e maior AEC/ACT pós-HD foram associados a pior sobrevida em um ano.

## Background

Volume excess and volume depletion are two major concerns in hemodialysis (HD) patients^1^. The correct assessment of the dry weight (DW) and the amount of fluid to be removed in each dialysis session is based on clinical judgement, considering blood pressure (BP), edema, dyspnea, and the inter-dialytic weight gain. Such evaluation is based in trial and error^2^, and when the DW is overestimated the patient remains with excessive fluid and is subjected to long term complications (left ventricular hypertrophy, hypertension) and higher mortality risk^3–5^. When underestimated, patients may present intra-dialytic hypotension, cramps, and confusion^5^.

Bioimpedance analysis (BIA) is an important and non-invasive method to evaluate body composition, and has been validated in different populations, including dialysis patients^6–10^. BIA and phase angle (PhA) measurements provide information on volume distribution and nutritional status, improving evaluation on malnourishment, frailty and sarcopenia^11,12,13^.

Body composition changes with age, generally with less fat-free mass (FFM) and more fat mass (FM), especially visceral fat. This further modifies body water distribution, with elderly patients having increased extracellular water (ECW) to total body water (TBW) ratio^14^. Such differences can impact the DW determination in older individuals and contribute to the short and long term complications mentioned above.

There are various definitions for excess volume, some with complex equations^4,7^. Adjusted ECW to TBW ratio is a validated index that has been associated with survival^15^. Nongnuch et al.^16^ considered two standard deviations from post-dialysis ECW/TBW to define excess fluid (≥ 0.41) and other authors encountered significance in ratios ≥ 0.47^17^.

## Aim

This study aimed to describe a sample of dialysis patients from a single center on their demographics, bioimpedance assessment of volume distribution and nutritional status, as well as mortality during a 12-month follow-up.

## Subjects and Methods

*Study design*: this was a prospective observational cohort study to evaluate the volume distribution and nutritional status of vintage young and elderly dialysis patients through BIA, clinical evaluation, anthropometric measurements, and laboratory data. The study was approved by the local Ethics Committee (approval number 2.494.773), and patients provided written informed consent prior to enrollment.

*Subjects*: Patients were eligible to participate if they were over 18 years old and had received maintenance HD 3 times per week for at least 3 months. Exclusion criteria were contraindications for BIA including pacemakers or limb amputations, acute illness, and unwillingness to participate.

*Clinical evaluation*: As usual, patients were clinically evaluated on symptoms of volume excess or depletion and blood pressure (BP), pre-dialysis weight, interdialytic weight gain (IDWG), and prescribed ultrafiltration (UF) volume. Intra-dialytic events were recorded. BP and body weight were recorded at the beginning and at the end of the session. Recorded values of BP and body weight in the preceding and following weeks were collected from the patients’ charts to ensure that the values on the day of BIA evaluation were equivalent to the other days and not unusually above or below the patients’ normal values. The same scale and sphygmomanometer were used for all patients.

*Bioimpedance evaluation*: BIA was conducted immediately before the dialysis session and 30 minutes after the end of the same session. Patients were in the supine position, and electrodes were placed in the arm without vascular access with BIA 450^TM^ Bioimpedance Analyzer (Biodynamics Corporation, USA) single-frequency device (50 kHz). Resistance, reactance, phase angle (PhA), body cell mass (BCM), fat mass (FM), lean mass (LM), body mass index (BMI), total body water (TBW), intracellular water (ICW), and extracellular water (ECW) values were recorded.

*Additional data*: Clinical and demographic characteristics were recorded, as well as anti-hypertensive medications in use. Routine blood exams collected at the first week of the month were evaluated to assess nutritional status, blood cell count, albumin, creatinine, C reactive protein, and electrolytes. Hand grip strength (HGS) was assessed using Crown Manual Dynamometer^®^ preferably with the dominant hand without vascular access, 3 times, with the highest value being recorded. Clinical complications, hospitalizations and death were recorded in the following 12 months.

*Statistics*: Categorical variables are presented as number and %, and compared by chi-square and Fisher’s exact test. Continuous variables with normal distribution are presented as mean and standard deviation (SD), and compared by parametric tests. Variables with non-normal distribution are presented as median and 25–75^th^ interquartiles and compared by non-parametric tests. Correlation between continuous variables is expressed as r and compared by Pearson or Spearman (non-parametric) coefficients. Patient survival was assessed by Kaplan-Meier and differences compared by log-rank test. To estimate the magnitude of survival difference, we used Cox proportional hazards model, with 95% confidence interval (CI). Receiver operator characteristics (ROC) curve and area under the curve (AUC) analysis and Youden’s J statistic were used to estimate the optimal cut-off point for variables associated with death. Significant differences were considered when p < 0.05. All analyses were performed using SPSS^®^ v. 27.

## Results

A total of 82 patients were included in the analysis, 51.2% male, 53.6% Caucasian, 29.2% elderly (≥ 65 years old). Clinical, demographic and laboratorial data are presented in Table 1. Mean follow-up time was 12.6 ± 4.8 months.

**Table 1.** Clinical and demographic characteristics and laboratory data of dialysis patients

	Total patients(n = 82)	Age < 65(n = 58)	Age ≥ 65(n = 24)	P
Age, years^a^	52.5 ± 17.4	44.1 ± 12.9	72.6 ± 7.3	**< 0.0001**
Male Gender	42 (51.2%)	28 (48.2%)	14 (58.3%)	0.471
Caucasian	44 (53.6%)	30 (51.7%)	14 (58.3%)	**0.046**
Dialysis (months)^b^	40.5 (10.7–79.2)	46.5 (14.0–79.2)	30.0 (9.2–99.7)	0.742
CKD etiology				
Diabetes	10 (12.1%)	4 (6.8%)	6 (25%)	
Hypertension	22 (26.8%)	13 (22.4%)	9 (37.5%)	
CGN	24 (29.2%)	21 (36.2%)	3 (12.5%)	0.224
APKD	6 (7.3%)	4 (6.8%)	2 (8.3%)	
Obstructive	6 (7.3%)	5 (8.6%)	1 (4.1%)	
Other	14 (17.0%)	11 (18.8%)	3 (12.5%)	
Diabetes	16 (19.5%)	6 (10.3%)	10 (41.6%)	**0.002**
Hypertension	75 (91.4%)	52 (89.6%)	23 (95.8%)	0.667
Heart failure	14 (17.0%)	9 (15.5%)	5 (20.8%)	0.538
Anti-HTN drugs				
None	13 (15.8%)	9 (15.5%)	4 (16.6%)	
1	17 (20.7%)	11 (18.9%)	6 (25%)	
2	29 (35.3%)	22 (37.9%)	7 (29.1%)	0.955
3	14 (17.0%)	10 (17.2%)	4 (16.6%)	
> 4	9 (10.9%)	6 (10.3%)	3 (12.5%)	
Beta-blockers	36 (43.9%)	30 (51.7%)	6 (25%)	**0.030**
ACEi/ARB	32 (39.0%)	27 (46.5%)	5 (20.8%)	**0.046**
Calcium channel inhibitors	31 (37.8%)	22 (37.9%)	9 (37.5%)	1.000
Vasodilators	14 (17.0%)	11 (18.9%)	3 (12.5%)	0.748
Diuretics	33 (40.2%)	23 (39.6%)	10 (41.6%)	1.000
BMI (kg/m)^a^	26.6 ± 6.0	26.5 ± 6.5	26.8 ± 4.9	0.821
Obesity (BMI ≥ 25)	46 (56%)	30 (51.7%)	16 (66.6%)	0.218
Dry weight (clinical), kg^b^	63 (51.8–85.2)	62.2 (51.8–86.0)	63.0 (51.8–83.7)	0.976
SBP (mmHg)^a^ pre-HD	142.1 ± 29.0	143.5 ± 27.4	138.6 ± 33.1	0.491
DBP (mmHg)^a^ pre-HD	75.7 ± 21.4	80.2 ± 21.6	65.0 ± 16.6	**0.003**
Hand grip strength^b^	18 (15–25)	18 (15–24)	17 (13–26)	0.653
Male^b^	25 (18–32)	15 (18–36.5)	23 (17–30)	0.250
Female^b^	15 (13–18)	16 (13–18.5)	12 (10–15)	**0.017**
Hemoglobin (g/dL)^b^	10.5 (9.8–11.3)	10.4 (9.3–11.3)	10.9 (9.9–11.4)	0.327
Ferritin (ng/dL)^b^	336 (190–562)	328 (179–530)	363 (207–853)	0.637
Creatinine (mg/dL)^a^	9.8 ± 2.9	10.5 ± 2.9	8.4 ± 2.2	**0.004**
Ionized Calcium (mg/dL)^b^	4.6 (4.3–4.8)	4.6 (4.2–4.8)	4.6 (4.3–4.8)	0.328
Phosphate (mg/dL)^a^	5.5 ± 1.5	5.4 ± 1.6	5.8 ± 1.4	0.328
Albumin (mg/dL)^a^	3.7 ± 0.3	3.7 ± 0.3	3.8 ± 0.3	0.708
Cholesterol (mg/dL)^b^	141 (117–170)	134 (115–162)	146 (125–185)	0.326
Triglycerides (mg/dL)^b^	125 (88–182)	125 (88–181)	125 (88–195)	0.788
HDL (mg/dL)^b^	39 (31–49)	38 (28–49)	39 (34–47)	0.937
LDL (mg/dL)^b^	78 (59–93)	72 (57–90)	81 (69–106)	0.321
C Reactive protein (mg/L)^b^	7.7 (1.8–16.1)	5.5 (1.4–16.0)	8.1 (2.2–20.0)	0.580

CKD: chronic kidney disease; CGN: chronic glomerulonephritis; APKD: adult polycystic kidney disease; anti-HTN: anti-hypertensive; ACEi: angiotensin-converting enzyme inhibitor; ARB: angiotensin II receptor blocker; BMI: body mass index; HD: hemodialysis; SBP: systolic blood pressure; DBP: diastolic blood pressure; HDL: high-density lipoproteins; LDL: low-density lipoproteins. ^a^mean ± standard deviation; ^b^median (IQ 25–75).

Body weight, SBP, and DBP were compared before and after dialysis on the day of BIA evaluation (D0) with the preceding (D-1, D-2, D-3) and following (D+1, D+2, D+3) days, and no significant differences in median values for all variables were observed (data not shown).

**Figure 1.**
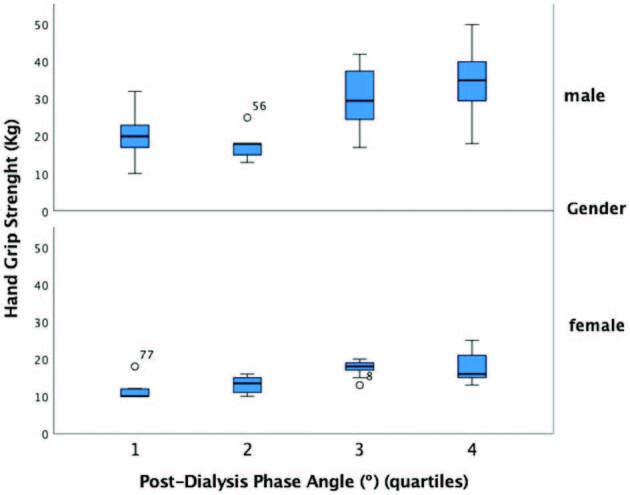
Hand grip strength and post-dialysis phase angle quartiles, according to gender.

Table 1 shows that there were more elderly patients with diabetes (41.6% vs. 10.3%, p = 0.002) and fewer elderly were prescribed beta-blockers (25% vs. 51.7%, p = 0.030) and ACE inhibitors or angiotensin receptor blockers (20.8% vs. 46.5%, p = 0.046). Elderly patients had significantly lower pre-HD DBP (65.0 ± 16.6 vs. 80.2 ± 21.6 mmHg, p = 0.003). Pre-HD DBP was not significantly different in patients with or without underlying heart failure (HF) (70.5 ± 30.1 vs. 76.8 ± 19.2 mmHg, p = 0.316). Except for lower creatinine levels (8.4 ± 2.2 vs. 10.5 ± 2.9 mg/dL, p = 0.004), there were no statistical differences in laboratory data regarding nutritional status between young and elderly patients. HGS was comparable between age groups overall and significantly lower in older female patients (Figure 1).

### BIA Results

Table 2 shows BIA assessments before and after HD in young and elderly patients.

**Table:** Body composition, fluid status evaluation, and blood pressure before and after hemodialysis session in young and elderly patients

	Total patients (n = 82)	Age < 65 (n = 58)	Age ≥ 65 (n = 24)	P
**PRE-DIALYSIS**				
Weight (kg)^a^	64.3 (54.6–88.1)	63.5 (54.6–88.4)	64.3 (54.3–87.4)	0.930
Lean Mass (kg)^a^	47.2 (38.7–60.3)	47.2 (38.7–63.9)	45.7 (39.4–53.7)	0.404
Fat Mass (kg)^a^	18.5 (12.5–28.3)	17.1 (11.2–28.1)	20.6 (14.7–29.5)	0.422
FFM (kg)^a^	47.2 (38.7–60.3)	47.2 (38.7–63.9)	45.7 (39.5–53.7)	0.384
TBW (L)^a^	33.6 (28.4–43.3)	34.1 (28.4–44.1)	33.2 (28.4–39.8)	0.429
ECW (L)^a^	17.2 (15.0–22.5)	16.7 (14.4–22.5)	18.0 (15.5–21.6)	0.531
ICW (L)^a^	16.4 (14.2–22.6)	16.8 (14.9–24.4)	14.8 (12.8–19.2)	**0.037**
TBW/FFM^a^	0.72 (0.71–0.74)	0.72 (0.71–0.75)	0.73 (0.71–0.74)	0.801
ICW/total body weight^a^	0.26 (0.22–0.29)	0.27 (0.23–0.31)	0.22 (0.21–0.25)	**< 0.0001**
ECW/TBW^b^	0.50 ± 0.05	0.48 ± 0.05	0.53 ± 0.03	**< 0.0001**
UF, prescribed (L)^b^	2.6 ± 1.6	2.8 ± 1.5	2.1 ± 1.5	0.087
Phase Angle (º)^b^	5.0 ± 1.12	5.3 ± 1.0	4.3 ± 0.9	**< 0.0001**
**POST-DIALYSIS**				
Weight (kg)^a^	63.0 (52.2–85.9)	62.6 (52.2–86.1)	63.0 (51.6–84.6)	0.964
Lean Mass (kg)^a^	42.7 (34.8–54.7)	43.2 (35.5–57.7)	42.0 (34.3–51.5)	0.535
Fat Mass (kg)^a^	21.1 (14.6–30.3)	19.3 (14.0–29.4)	22.5 (17.9–32.5)	0.458
FFM (kg)^a^	42.3 (34.8–54.7)	42.7 (35.5–53.7)	42.0 (34.3–51.5)	0.553
TBW (L)^a^	31.3 (26.6–38.8)	31.5 (26.7–40.4)	30.6 (25.8–37.4)	0.483
ECW (L)^a^	15.1 (12.5–17.8)	14.3 (11.8–18.2)	15.8 (13.0–17.8)	0.209
ICW (L)^a^	16.2 (14.3–21.6)	16.5 (14.8–23.2)	14.7 (12.3–18.6)	**0.018**
TBW/FFM^a^	0.73 (0.71–0.76)	0.74 (0.71–0.76)	0.73 (0.71–0.75)	0.532
ICW/total body weight^b^	0.26 ± 0.05	0.27 ± 0.05	0.23 ± 0.02	**< 0.0001**
ECW/TBW^b^	0.46 ± 0.06	0.44 ± 0.05	0.51 ± 0.04	**< 0.0001**
ECW/TBW ≥ 0.47	41 (50%)	21 (36.2%)	20 (83.3%)	**< 0.0001**
UF, net (L)^b^	2.3 ± 1.2	2.5 ± 1.2	1.9 ± 1.1	**0.039**
Phase Angle (^o^)^b^	6.0 ± 1.58	6.4 ± 1.4	4.9 ± 1.3	**< 0.0001**
Reached prescribed dry weight	68 (82.9%)	48 (82.7%)	20 (83.3%)	1.000

BIA: bioimpedance analysis. TBW: total body water. ICW: intracellular water. ECW: extracellular water. FFM: fat free mass. UF: ultrafiltration. SBP: systolic blood pressure. DBP: diastolic blood pressure. ^a^median (IQ 25–75); ^b^mean ± standard deviation.

The evaluation performed before HD demonstrated that elderly patients had lower PhA (4.3 ± 0.9 vs. 5.3 ± 1.0º, p < 0.0001), lower ICW [14.8 (12.8–19.2) vs. 16.8 (14.9–24.4) L, p = 0.037], lower ICW to total body weight ratio (0.23 ± 0.02 vs. 0.27 ± 0.05, p < 0.0001), and higher ECW/TBW (0.53 ± 0.03 vs. 0.48 ± 0.05, p < 0.0001). These differences remained significant in the post-HD measurements (Table 2).

Comparing pre- and post-HD BIA measurements, there was no significant change in ICW [16.4 (14.2–30.3) vs. 16.2 (14.3–21.6) L, p = 0.52)]. There was a significant reduction in ECW [17.1 (14.9–22.3) vs. 15.2 (12.5–17.8) L, p < 0.0001)], lean mass (47.2 vs. 42.7 kg, p = 0.022) and ECW/TBW (0.49 ± 0.05 vs. 0.46 ± 0.06, p < 0.0001) (Table 3).

**Table 3.** ANOVA of pre- and post-dialysis body composition, phase angle, and blood pressure assessments

	Pre-HD n = 82	Post-HD n = 82	P
Weight (kg)^a^	64.3 (54.6–99.1)	63.0 (52.2–85.9)	0.451
TBW (L)^a^	33.6 (28.4–43.4)	31.3 (26.6–38.8)	**0.021**
ECW (L)^a^	17.1 (14.9–22.3)	15.1 (12.5–17.8)	**< 0.0001**
ICW (L)^a^	16.4 (14.2–30.3)	16.2 (14.3–21.6)	0.528
Lean Mass (kg)^a^	47.2 (38.7–60.3)	42.7 (34.8–54.7)	**0.022**
Fat Mass (kg)^a^	18.5 (12.5–28.3)	21.1 (14.6–22.6)	0.156
FFM (kg)^a^	47.2 (38.7–60.3)	42.3 (34.8–54.7)	**0.020**
TBW/FFM^b^	0.73 ± 0.03	0.74 ± 0.04	0.065
ICW/total body weight^a^	0.25 (0.22–0.29)	0.25 (0.22–0.30)	0.710
ECW/TBW^b^	0.49 ± 0.05	0.46 ± 0.06	**< 0.0001**
ECW/TBW ≥ 0.47	58 (70.7%)	41 (50%)	**< 0.0001**
Phase Angle (º)^b^	5.0 ± 1.1	6.0 ± 1.5	**< 0.0001**
SBP (mmHg)^b^	141.8 ± 30.7	138.8 ± 28.2	0.512
DBP (mmHg)^b^	76.3 ± 21.4	74.3 ± 17.1	0.505

BIA: bioimpedance analysis. TBW: total body water. ECW: extracellular water. ICW: intracellular water. FFM: fat free mass. SBP: systolic blood pressure. DBP: diastolic blood pressure. ^a^median (IQ 25–75); ^b^mean ± standard deviation.

### Nutritional Assessments

PhA was directly correlated with albumin (r = 0.286, p = 0.009), creatinine (r = 0.409, p < 0.0001), and HGS (r = 0.471, p < 0.0001), and inversely correlated with ferritin (r = –0.230, p = 0.038), C reactive protein (r = –0.319, p = 0.01), age (r= –0.439, p < 0.0001), and ECW/TBW (r= –0.829, p < 0.0001).

PhA increased significantly when comparing pre- and post-HD measurements (5 ± 1.1 vs. 6 ± 1.5º, p < 0.001). Table 4 presents post-HD PhA in quartiles, demonstrating a significant difference in age, C reactive protein and ECW/TBW (inverse relationship), and in creatinine, albumin, and ICW (direct relationship). There was no difference regarding race, gender, hypertension, diabetes, time in dialysis, BMI, ICW/total body weight (data not shown).

**Table 4.** Post-dialysis phase angle quartiles and body composition and laboratorial results

	Phase Angle quartiles				P
	Q1 (≤ 4.9) n = 21	Q2 (4.9–5.9) n = 21	Q3 (5.9–7.3) n = 21	Q4 (≥ 7.3) n = 19	
Age, years^b^	64.8 ± 16.0	48.6 ± 17.9	52.0 ± 12.9	43.4 ± 15.5	**< 0.0001**
Creatinine (mg/dL)^b^	8.9 ± 2.2	8.9 ± 2.4	10.1 ± 2.5	11.5 ± 3.6	**0.013**
Albumin (mg/dL)^b^	3.6 ± 0.3	3.7 ± 0.3	3.8 ± 0.3	3.9 ± 0.2	**0.020**
C Reactive protein (mg/L)^a^	20 (8.1–28.1)	7.5 (1.6–17.8)	4.4 (1.9–13.7)	3.8 (0.5–13.2)	**0.019**
TBW (L)^a^	30 (26.2–37.4)	28 (25.2–33.3)	32.1 (27.8–42.1)	35.8 (27.3–42.1)	0.268
ECW (L)^a^	15.9 (14–19.8)	13.4 (11.9–15.6)	15.6 (12.5–19.1)	13.7 (10.8–16.5)	0.056
ICW (L)^a^	14.5 (12–17.6)	14.9 (13.2–17.0)	17.5 (15.1–22.9)	21.6 (15.6–25.3)	**< 0.0001**
Lean Mass (kg)^a^	41.9 (35–52.7)	36.9 (32.8–46.7)	44.1 (37.1–59.2)	49.9 (36.4–57.2)	0.271
Fat Mass (kg)^a^	19.9 (14–29)	18.5 (11.2–30.5)	27.9 (18.4–31.5)	18.6 (15–29.6)	0.419
TBW/FFM^a^	0.73 (0.71–0.75)	0.74 (0.72–0.76)	0.73 (0.71–0.76)	0.71 (0.70–0.75)	0.530
ECW/TBW^b^	0.53 ± 0.04	0.47 ± 0.03	0.45 ± 0.03	0.39 ± 0.04	**< 0.0001**
ECW/TBW ≥ 0.47	20 (95%)	13 (61.9%)	7 (33.3%)	1 (5.2%)	**< 0.0001**
Phase Angle (º)^b^	4 ± 0.6	5.5 ± 0.3	6.6 ± 0.4	8 ± 0.8	< 0.0001

TBW: total body water. ECW: extracellular water. ICW: intracellular water. FFM: fat free mass. ^a^median (IQ 25–75); ^b^mean ± standard deviation.

### Excess Fluid, Blood Pressure and Mortality

Most patients (83%) were considered to reach their clinically prescribed DW. As shown above, elderly patients had higher ECW/TBW, before and after HD.

Fifteen patients (18.2%) died during follow-up, two from neoplasms and the remaining from cardiovascular disease. Mean follow-up time for cardiovascular death was 7.6 ± 4.0 months. Eight (53%) of the deceased patients were elderly.

Death was significantly associated with age (62.6 ± 17.8 vs. 50.2 ± 16.6 years, p = 0.012), pre-HD PhA (4.3 ± 1.3 vs. 5.2 ± 1.0º, p = 0.004), post-HD PhA (4.8 ± 1.7 vs. 6.2 ± 1.4º, p = 0.0001), and post-HD ECW/TBW (0.50 ± 0.05 vs. 0.45 ± 0.06, p = 0.015). Pre-HD ECW/TBW was not significantly associated with death (0.52 ± 0.05 vs. 0.49 ± 0.05, p = 0.055). Death was associated with lower albumin (3.6 ± 0.3 vs. 3.8 ± 0.3 mg/dL, p = 0.021), lower creatinine (8.1 ± 1.9 vs. 10.2 ± 2.9 mg/dL, p = 0.012), and higher ferritin [462.0 (40–2682) vs 334.0 (18–1414) ng/dL, p = 0.019].

**Figure 2.**
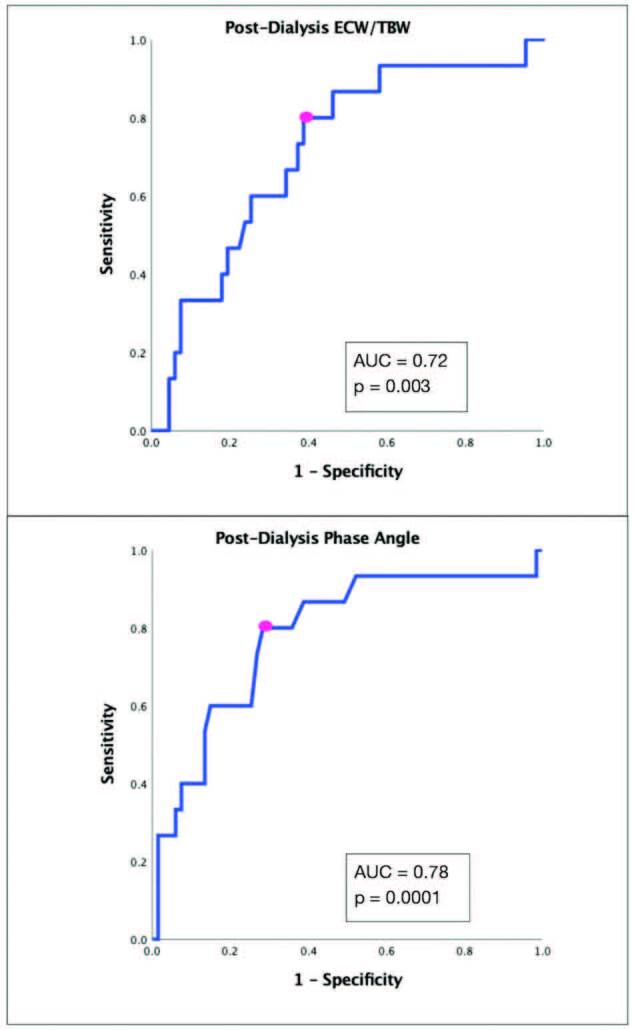
Receiver operator characteristic (ROC) curve for mortality prediction. TOP: Post-dialysis extracellular water to total body water ratio (ECW/TBW). BOTTOM: Post-dialysis phase angle. AUC: area under the curve.

Receiver operating characteristic curve (ROC curve) evaluation for post-HD ECW/TBW in predicting mortality was 0.72 (95% CI 60.9–81.3), p = 0.003. The cut-off value of ≥ 0.47 provided 80% sensitivity (95% CI 51.9–95.7) with 61.2% specificity (95% CI 48.5–72.9), with a Youden’s J score of 0.41. Positive predictive value (PPV) was 31.6% (95% CI 23.8–40.6) and negative predictive value (NPV) was 93.2% (95% CI 83.0–97.5) (Figure 2).

ROC curve evaluation for post-HD PhA to predict mortality was 0.78 (95% CI 67.7–86.6), p = 0.0001. The cut-off value of ≤ 5.5º provided 80% sensitivity (95% CI 51.9–95.7) with 71.6% specificity (95% CI 59.3–82.0), with a Youden’s J score of 0.51. PPV was 38.7% (95% CI 28.6–49.9) and NPV was 94.1% (95% CI 85.2–97.8) (Figure 2).

Survival was lower for patients with post-HD ECW/TBW ≥ 0.47 (69.5% vs. 90.6%, p = 0.019), for elderly patients (64.2%, vs. 86.2%, p = 0.029), and for patients with lower post-HD PhA quartiles (q1 = 55.5% vs. q2 = 79.2% vs. q3 = 93.3% vs. q4 = 93.8%, p = 0.006). Cox regression analysis demonstrated that only PhA [HR 5.04 (CI 95% 1.60–15.86), p = 0.006] remained associated with death after adjusting for age, ECW/TBW, race, heart failure, obesity, BMI, diabetes, hypertension, and HDL-cholesterol.

**Figure 3.**
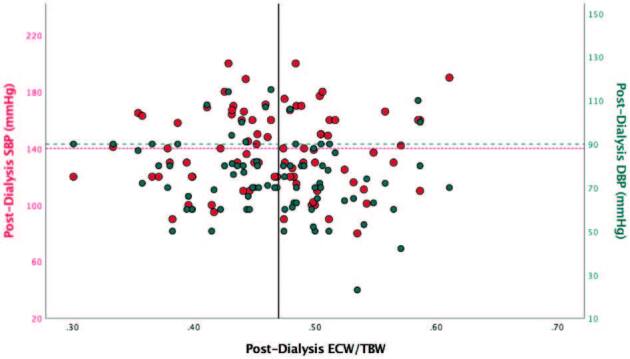
Scatter plot of post-dialysis extracellular water to total body water ratio (ECW/TBW) distribution for post-dialysis systolic and diastolic blood pressure (SBP, DBP).

Excess fluid was not significantly associated to systolic or diastolic hypertension. Figure 3 demonstrates that 55% (22/40) of patients with ECW/TBW ≥ 0.47 had systolic blood pressure (SBP) < 140 mmHg and 85% (34/40) had diastolic blood pressure (DBP) < 90 mmHg. Conversely, 54% (23/42) of patients without excess fluid had SBP ≥ 140 mmHg and 26% (11/42) had DBP ≥ 90 mmHg.

## Discussion

We presented the characteristics of a sample of prevalent adult HD patients from a single-center and compared the volume distribution and nutritional status of young and elderly groups through BIA. In summary, our study demonstrated that ICW was significantly lower and ECW/TBW was significantly higher in elderly patients. Most patients had excess fluid, and post-HD ECW/TBW ≥ 0.47 was associated with worse survival. PhA was narrower in older patients, and was correlated with nutritional parameters and with lower survival.

BIA is considered a safe and reliable tool for evaluating body composition and water distribution, and has been validated in HD patients^1,9^. Various equations have been proposed to define excess fluid and enable comparisons and correlations with clinical outcomes^7,8,18^, but the differences between equipment and populations are still a challenge, requiring internal validation. Hence, raw data and ratios are an interesting way to present the BIA findings, and repeating measurements over time may yield more useful information on the patient level.

Body composition changes with age, with a reduction in muscle and lean tissue mass and an increase in fat mass. ICW measurements are used as a close approximation for body cell mass, an important parameter for assessing nutritional status^19^. In the present study, elderly patients had lower ICW than younger patients.

In a cross-sectional study, Lee et al.^14^ compared young and elderly HD patients using BIA, and their findings regarding body composition and PhA are comparable to ours. They demonstrated higher ECW/TBW in older subjects and argued that this could be explained by more excess fluid and poorer nutritional status in such patients.

Excess fluid is a known risk factor for mortality in HD patients^4^, and clinical probing of dry weight rely mainly on BP, edema, and dyspnea. Most patients with excess fluid have higher BP, but not all hypertensive patients have fluid overload^20^. In the elderly population, arterial stiffness may elevate SBP, and cardiac disease may decrease diastolic values. Thus, BP control alone may be a misleading surrogate of fluid status in this population.

Perez-Morales et al.^17^ recently published a proof-of-concept study that found ECW/TBW ≥ 0.47 to be associated with higher risk of mortality using ROC curve analysis. In the present study, ECW/TBW ≥ 0.47 was also the cut-off value with best sensitivity and specificity to predict mortality during follow-up.

Most (83%) elderly patients in our study had post-HD ECW/TBW ≥ 0.47. Elderly patients had lower pre-HD DPB and 40% were diabetic. One third of the younger patients also had excess fluid after dialysis. Castellano et al.^21^ identified two subsets of patients in which achieving volume balance was especially difficult. One subset was of co-morbid diabetic males that used a large number of antihypertensive drugs. The other was of nondiabetic young patients who did not comply with treatment recommendations. Abbas et al.^10^ found that diabetic patients had significantly lower efficiency of removing fluid during dialysis, possibly due to impaired vascular refilling.

Although it is well established that the maximum ultrafiltration rate should not be greater than 12 mL/kg/h^22,23^, several patients had an excessive interdialytic weight gain, demanding higher volumes of fluid removal in one HD session. Salt and water restrictions are a very important part of treatment, but many patients struggle to follow the dietary prescription^24,25^. Socioeconomic status, employment, and formal education also contribute to non-adherence to dietary guidance^26,27^. Ultra-processed food, rich in salt and additives account for a significant portion of patients’ daily intake^28^. Although we did not perform a socioeconomic questionnaire, we recognize that most patients at our facility have low income, are undereducated, rely on the public health care system, and receive social security benefits. The proportion of overweight and obese patients and of hyperphosphatemic patients possibly reflect the consumption of ultra-processed food.

Bioimpedance-guided fluid management has been associated with better volume and BP control^29,30^, decreased arterial stiffness and left ventricular mass index,^29^ and survival benefits^1,31^. Wabel et al.^20^ classified patients into groups according to their SBP and fluid status, and described that grossly “overhydrated” (OH) (determined by mass of excess fluid [MExF] > 2.5L) patients were more unlikely to reach “normohydration” by the end of HD. The authors argued that normotensive “overhydrated” patients may not be adequately treated because they are perceived as “normohydrated” or because they are more likely to present symptoms of volume depletion. These symptoms may be due to antihypertensive medication use, underlying heart disease, or even hypoalbuminemia. Low SBP is also associated with mortality^32^, possibly reflecting cardiac insufficiency. Some hypertensive patients may actually have reached their volume balance and cannot improve their BP control with more ultrafiltration. In the present study, excess fluid was not associated with systolic or diastolic hypertension, with a great proportion of patients considered to have excess fluid but normotensive or with adequate volume and hypertensive.

PhA is a measurement associated with cell membrane integrity and vitality. PhA correlates to nutritional status and muscle strength^33^. Beberashvili et al.^33^ demonstrated that lower PhA tertiles were associated with increased morbidity and decreased survival in HD patients. PhA is narrower with increasing age, and in women compared to men^34^. In the present study, PhA was narrower in older patients and significantly correlated with nutritional parameters, such as albumin, creatinine, and HGS. PhA was also directly correlated to ICW and inversely correlated to ECW/TBW, and lower quartiles were associated with higher mortality. Post-HD PhA measurements are more accurate, as fluid and electrolytes are expected to be more balanced^35^.

In a systematic review, Tabinor et al.^36^ analyzed 42 cohorts of chronic kidney failure patients. In 31 cohorts, excess fluid was independently associated with all-cause and cardiovascular mortality. They also performed a subgroup meta-analysis with 12 cohorts that reported multivariate analyses with similar cut-off values, and found that a one degree decrease in PhA and higher excess fluid were both predictors of mortality, almost doubling the risk.

More recently, Wang and Gu^37^ conducted a meta-analysis involving 55 studies with 104,758 HD patients. There was an increased risk of mortality with ECW/TBW > 0.4 (HR 5.912, 95% CI: 2.016–17.342), ECW/ICW for every 0.01 increase (HR 1.041, 95% CI: 1.031–1.051), and MExF/ECW > 15% (HR 2.722, 95% CI: 2.005–3.439). A one-degree increase in PhA was a protective factor for both mortality (HR 0.676, 95% CI: 0.474–0.879) and cardiovascular events (HR 0.736, 95% CI: 0.589–0.920).

These findings are in consistent with our report, demonstrating that BIA assessments of ECW/TBW and PhA can yield important and useful information that may impact patient care.

This study has some limitations. First, the small sample size may have limited the ability to demonst- rate statistical significance in some aspects and does not permit extrapolation of our findings to other populations. Second, we analyzed only a baseline and not serial measurements, which could have been valuable for understanding the association of ECW/TBW and PhA and survival. The strength of this study is the large compilation of raw data, separating young and elderly patients, before and after HD, and serves as a basis for further studies to clarify the relationship between age, volume distribution, and nutritional parameters of HD patients.

In conclusion, BIA is a useful tool for assessing volume distribution and nutrition in HD patients. It is a simple and reproducible evaluation, and may help determine optimal dry weight together with clinical judgement, especially in elderly patients. Narrower PhA and higher ECW/TBW after HD were associated with poorer one-year survival.

## References

[1] Onofriescu M, Hogas S, Voroneanu L, Apetrii M, Nistor I, Kanbay M (2014). Bioimpedance-guided fluid management in maintenance hemodialysis:
a pilot randomized controlled trial. Am J Kidney Dis..

[2] Wabel P, Chamney P, Moissl U, Jirka T. (2009). Importance of whole-body bioimpedance spectroscopy for the
management of fluid balance. Blood Purif..

[3] Tonelli M, Wiebe N, Culleton B, House AA, Rabbat C, Fok M (2006). Chronic kidney disease and mortality risk: a systematic
review. J Am Soc Nephrol..

[4] Wizemann V, Wabel P, Chamney P, Zaluska W, Moissl U, Rode C (2009). The mortality risk of overhydration in haemodialysis
patients. Nephrol Dial Transplant..

[5] Passauer J, Petrov H, Schleser A, Leicht J, Pucalka K. (2010). Evaluation of clinical dry weight assessment in haemodialysis
patients using bioimpedance spectroscopy: a cross-sectional
study. Nephrol Dial Transplant..

[6] Basile C, Vernaglione L, Di Iorio B, Bellizzi V, Chimienti D, Lomonte C (2007). Development and validation of bioimpedance analysis prediction
equations for dry weight in hemodialysis patients. Clin J Am Soc Nephrol..

[7] Chamney PW, Wabel P, Moissl UM, Müller MJ, Bosy-Westphal A, Korth O (2007). A whole-body model to distinguish excess fluid from the hydration
of major body tissues. Am J Clin Nutr..

[8] Chazot C, Wabel P, Chamney P, Moissl U, Wieskotten S, Wizemann V. (2012). Importance of normohydration for the long-term survival of
haemodialysis patients. Nephrol Dial Transplant..

[9] Covic A, Ciumanghel AI, Siriopol D, Kanbay M, Dumea R, Gavrilovici C (2017). Value of bioimpedance analysis estimated “dry weight” in
maintenance dialysis patients: a systematic review and
meta-analysis. Int Urol Nephrol..

[10] Abbas SR, Thijssen S, Penne EL, Raimann JG, Liu L, Sipahioglu MH (2018). Effect of change in fluid status evaluated by bioimpedance
techniques on body composition in hemodialysis patients. J Ren Nutr..

[11] Saitoh M, Ogawa M, Kondo H, Suga K, Takahashi T, Itoh H (2020). Bioelectrical impedance analysis-derived phase angle as a
determinant of protein-energy wasting and frailty in maintenance
hemodialysis patients: retrospective cohort study. BMC Nephrol..

[12] Macedo C, Amaral TF, Rodrigues J, Santin F, Avesani CM. (2021). Malnutrition and sarcopenia combined increases the risk for
mortality in older adults on hemodialysis. Front Nutr..

[13] Zhou H, Yao W, Pan D, Sun G. (2021). Predicational ability of phase angle on protein energy wasting in
kidney disease patients with renal replacement therapy: a cross-sectional
study. Food Sci Nutr..

[14] Lee JE, Jo IY, Lee SM, Kim WJ, Choi HY, Ha SK (2015). Comparison of hydration and nutritional status between young and
elderly hemodialysis patients through bioimpedance analysis. Clin Interv Aging..

[15] Davies SJ, Davenport A. (2014). The role of bioimpedance and biomarkers in helping to aid
clinical decision-making of volume assessments in dialysis
patients. Kidney Int..

[16] Nongnuch A, Panorchan K, Davenport A. (2014). Predialysis NTproBNP predicts magnitude of extracellular volume
overload in haemodialysis patients. Am J Nephrol..

[17] Perez-Morales R, Donate-Correa J, Martin-Nunez E, Perez-Delgado N, Ferri C, Lopez-Montes A (2021). Extracellular water/total body water ratio as predictor of
mortality in hemodialysis patients. Ren Fail..

[18] Guo Q, Lin J, Li J, Yi C, Mao H, Yang X (2015). The Effect of fluid overload on clinical outcome in southern
Chinese patients undergoing continuous ambulatory peritoneal
dialysis. Perit Dial Int..

[19] Çelik G, Oc B, Kara I, Yılmaz M, Yuceaktas A, Apiliogullari S. (2011). Comparison of nutritional parameters among adult and elderly
hemodialysis patients. Int J Med Sci..

[20] Wabel P, Moissl U, Chamney P, Jirka T, Machek P, Ponce P (2008). Towards improved cardiovascular management: the necessity of
combining blood pressure and fluid overload. Nephrol Dial Transplant..

[21] Castellano S, Palomares I, Molina M, Perez-Garcia R, Aljama P, Ramos R (2014). Clinical, analytical and bioimpedance characteristics of
persistently overhydrated haemodialysis patients. Nefrologia..

[22] Movilli E, Gaggia P, Zubani R, Camerini C, Vizzardi V, Parrinello G (2007). Association between high ultrafiltration rates and mortality in
uraemic patients on regular haemodialysis: a 5-year prospective
observational multicentre study. Nephrol Dial Transplant..

[23] Flythe JE, Kimmel SE, Brunelli SM. (2011). Rapid fluid removal during dialysis is associated with
cardiovascular morbidity and mortality. Kidney Int..

[24] Beerappa H, Chandrababu R. (2019). Adherence to dietary and fluid restrictions among patients
undergoing hemodialysis: an observational study. Clin Epidemiol Glob Health..

[25] Opiyo RO, Nyasulu PS, Olenja J, Zunza M, Nguyen KA, Bukania Z (2019). Factors associated with adherence to dietary prescription among
adult patients with chronic kidney disease on hemodialysis in national
referral hospitals in Kenya: a mixed-methods survey. Renal Replacement Therapy..

[26] Nerbass FB, Correa D, Santos RGD, Kruger TS, Sczip AC, Vieira MA (2017). Perceptions of hemodialysis patients about dietary and fluid
restrictions. J Bras Nefrol..

[27] Beerendrakumar N, Ramamoorthy L, Haridasan S. (2018). Dietary and fluid regime adherence in chronic kidney disease
patients. J Caring Sci..

[28] Wendling AL, Balbino KP, Ribeiro PVM, Epifânio APS, Marota LD, Hermsdorff HHM. (2020). Processed and ultra-processed food consumption are related to
metabolic markers in hemodialysis subjects. Rev Nutr..

[29] Hur E, Usta M, Toz H, Asci G, Wabel P, Kahvecioglu S (2013). Effect of fluid management guided by bioimpedance spectroscopy on
cardiovascular parameters in hemodialysis patients: a randomized controlled
trial. Am J Kidney Dis..

[30] Moissl U, Arias-Guillen M, Wabel P, Fontsere N, Carrera M, Campistol JM (2013). Bioimpedance-guided fluid management in hemodialysis
patients. Clin J Am Soc Nephrol..

[31] Kim YJ, Jeon HJ, Kim YH, Jeon J, Ham YR, Chung S (2015). Overhydration measured by bioimpedance analysis and the survival
of patients on maintenance hemodialysis: a single-center
study. Kidney Res Clin Pract..

[32] Dekker M, Konings C, Canaud B, Carioni P, Guinsburg A, Madero M (2018). Pre-dialysis fluid status, pre-dialysis systolic blood pressure
and outcome in prevalent haemodialysis patients: results of an international
cohort study on behalf of the MONDO initiative. Nephrol Dial Transplant..

[33] Beberashvili I, Azar A, Sinuani I, Shapiro G, Feldman L, Stav K (2014). Bioimpedance phase angle predicts muscle function, quality of
life and clinical outcome in maintenance hemodialysis
patients. Eur J Clin Nutr..

[34] Chertow GM, Lazarus JM, Lew NL, Ma L, Lowrie EG. (1997). Bioimpedance norms for the hemodialysis
population. Kidney Int..

[35] Ikizler TA, Burrowes JD, Byham-Gray LD, Campbell KL, Carrero JJ, Chan W (2020). KDOQI clinical practice guideline for nutrition in CKD: 2020
update. Am J Kidney Dis..

[36] Tabinor M, Elphick E, Dudson M, Kwok CS, Lambie M, Davies SJ. (2018). Bioimpedance-defined overhydration predicts survival in end stage
kidney failure (ESKF): systematic review and subgroup
meta-analysis. Sci Rep..

[37] Wang Y, Gu Z. (2021). Effect of bioimpedance-defined overhydration parameters on
mortality and cardiovascular events in patients undergoing dialysis: a
systematic review and meta-analysis. J Int Med Res..

